# Investigating the mediating role of learning engagement in the relationship between self-efficacy for managing emotional challenges and subjective well-being among medical students

**DOI:** 10.1038/s41598-026-40021-8

**Published:** 2026-02-17

**Authors:** Gholamreza Hamidkholgh, Erfan Zare, Alireza Mirzaei, Reza Nemati-Vakilabad, Mahzad Yousefian

**Affiliations:** 1 Students Research Committee, School of Medicine, Ardabil University of Medical Sciences, Ardabil, Iran; 2 Department of Emergency and Traditional Medicine, School of Medicine, Ardabil University of Medical Sciences, Ardabil, Iran; 3 Cancer Immunology and Immunotherapy Research Center, Ardabil University of Medical Sciences, Ardabil, Iran; 4 Department of Emergency Nursing, School of Nursing and Midwifery, Ardabil University of Medical Sciences, Ardabil, Iran; 5 Department of Medical-Surgical Nursing, School of Nursing and Midwifery, Ardabil University of Medical Sciences, Ardabil, Iran; 6 Department of anesthesiology, Fatemi hospital, School of Medicine, Ardabil University of Medical Sciences, Ardabil, Iran

**Keywords:** Self efficacy, Emotions, Learning, Engagement, Subjective, Well-being, Medical students, Education, Health care, Psychology, Psychology

## Abstract

Medical students often face significant emotional challenges that can adversely affect their subjective well-being and academic performance. Understanding the mechanisms that influence these outcomes is critical for fostering resilience and success in medical education. This study aims to investigate the mediating role of learning engagement in the relationship between self-efficacy to manage emotional challenges and subjective well-being among medical students. A cross-sectional study was conducted with 237 medical students (from various academic years) at Ardabil University of Medical Sciences, Iran, using validated questionnaires to measure self-efficacy, learning engagement, and subjective well-being. Data were analyzed using descriptive statistics, Pearson correlation coefficients, hierarchical linear regression, and path analysis. The findings revealed significant positive correlations among self-efficacy in managing emotional challenges, learning engagement, and subjective well-being. Mediation analysis showed that the direct effect of self-efficacy on subjective well-being remained significant in the presence of the mediator, learning engagement (b = 0.28, *p* < 0.001). Furthermore, the indirect effect of self-efficacy on subjective well-being through learning engagement was also significant (b = 0.22, *p* = 0.003), confirming that learning engagement partially mediates the relationship between self-efficacy and subjective well-being. Together, these variables accounted for 48% of the variance in subjective well-being. This study emphasizes the role of self-efficacy in managing emotional challenges and its positive effects on medical students’ learning engagement and well-being. Highlighting learning engagement as a key factor offers insights for educational planners. Targeted interventions to develop emotional management skills and active learning can boost academic performance and resilience. Future research should explore these findings across different cultures and use longitudinal designs to clarify causal relationships, enhancing support systems for medical students.

## Introduction

The influence of social and educational contexts on students is a vital area of examination. For medical students, the transition into clinical environments presents unique emotional challenges that can severely impact their development and well-being^1^. Medical students frequently encounter emotionally demanding situations during work-based learning, such as interacting with severely ill or complex patients, delivering bad news, being questioned in their clinical judgment, and navigating complex healthcare team dynamics^1^. These experiences can generate significant stress, self-doubt, and feelings of inadequacy, which are known risk factors for burnout and diminished well-being^2,3^.

In this context, the concept of self-efficacy, specifically a physician’s self-efficacy in managing emotional challenges (PSMEC), becomes critically relevant^4^. Grounded in Bandura’s social cognitive theory, self-efficacy refers to an individual’s belief in their capability to organize and execute courses of action required to manage prospective situations^5^. Unlike broader academic self-efficacy, physician self-efficacy to manage emotional challenges is a domain-specific construct that directly addresses the affective and interpersonal demands of clinical practice^1,4^. Recent research demonstrates that such self-efficacy beliefs are not only central to professional development and clinical decision-making but are also robustly associated with key adaptive outcomes^6,7^. Specifically, higher levels of self-efficacy in managing emotional challenges have been linked to greater resilience, lower levels of stress, anxiety, and depressive symptoms among medical trainees^8,9^.

A primary adaptive outcome linked to self-efficacy is learning engagement, the energy, dedication, and cognitive investment a student directs toward their educational activities^10–12^. According to social cognitive theory, individuals with strong self-efficacy beliefs are more likely to engage deeply, persist through difficulties, and employ effective cognitive strategies^13^. While the link between general academic self-efficacy and engagement is established^14,15^, the relationship between the emotion-focused self-efficacy required in clinical settings and medical students’ engagement in their learning remains underexplored^1,16^. It is plausible that students who believe they can cope with the emotional demands of clinical work are more likely to engage fully and constructively with their learning opportunities, rather than becoming avoidant or disengaged due to affective overload^17,18^.

Furthermore, both self-efficacy and learning engagement are posited to contribute to medical students’ subjective well-being^19,20^. Subjective well-being, encompassing life satisfaction and the relative presence of positive over negative affect, is a crucial marker of psychological health^21,22^. Emotional challenges are a significant threat to medical students’ well-being^1^. Therefore, a sense of efficacy in managing these challenges should directly foster greater competence and satisfaction (i.e., well-being)^23^. Concurrently, the pathway from self-efficacy to well-being may operate indirectly through learning engagement^15,22,24^. When students feel efficacious in handling emotional stressors, they are likely to engage more deeply with their training^25,26^. This active, positive engagement can itself be a source of fulfillment, mastery experiences, and positive affect, thereby enhancing overall subjective well-being^27^. This proposed mediation model aligns with theories suggesting that adaptive cognitive beliefs (self-efficacy) promote positive behavioral patterns (engagement), which in turn enhance emotional and evaluative states (well-being)^22^.

Significant gaps persist in understanding these dynamics, specifically within medical education^28^. While the PSMEC scale has been validated as a reliable measure of this domain-specific self-efficacy^1^, its associations with learning engagement and subjective well-being have not been empirically tested. Addressing this gap is essential for developing targeted interventions. If self-efficacy in managing emotional challenges promotes engagement and well-being, educational planners and support services can prioritize fostering this competency to reduce attrition, prevent burnout, and cultivate resilient, engaged future physicians.

Based on this rationale, the present study aims to investigate the relationships between physician self-efficacy to manage emotional challenges (PSMEC), learning engagement, and subjective well-being among medical students. We hypothesize that:

### H1:

 Self-efficacy to manage emotional challenges is positively associated with subjective well-being in medical students.

### H2:

 Self-efficacy to manage emotional challenges is positively associated with learning engagement in medical students.

### H3:

Learning engagement is positively associated with subjective well-being in medical students.

### H4:

 Learning engagement mediates the relationship between self-efficacy in managing emotional challenges and subjective well-being among medical students.

**Fig. 1:**
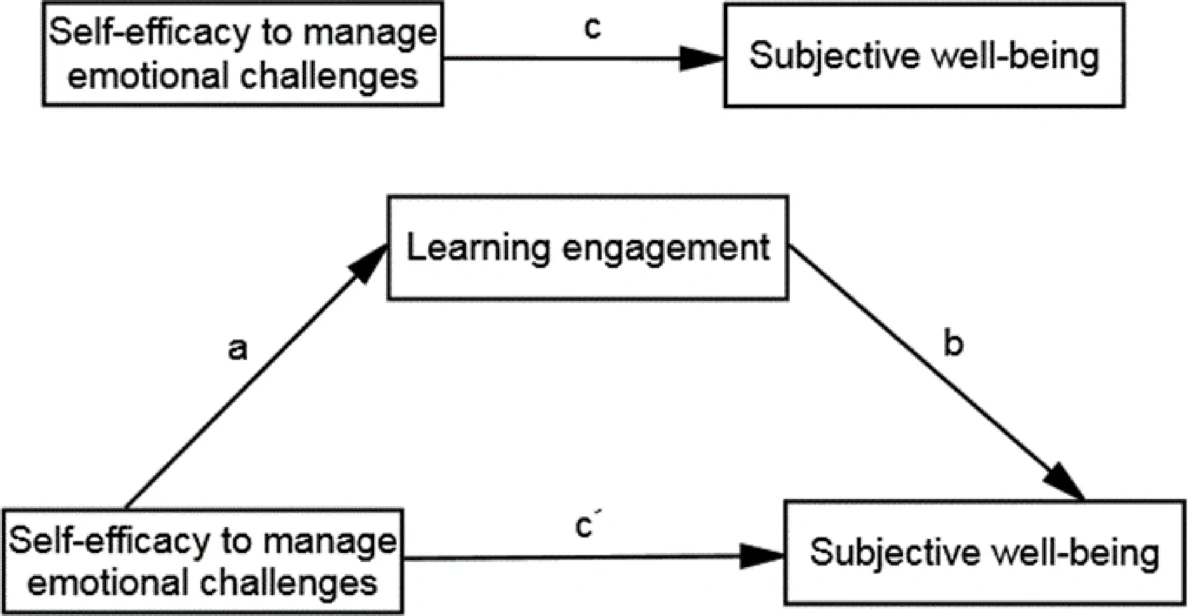
The hypothesized theoretical model of the study.

## Methods

### Design

This cross-sectional study was conducted and reported in accordance with the Strengthening the Reporting of Observational Studies in Epidemiology (STROBE) guidelines^28^.

### Setting and samples

This research was conducted among medical students at Ardabil University of Medical Sciences (ARUMS), Iran, from July to December 2024. The study population consisted of all medical students enrolled in the clinical phase of the 7-year medical doctorate program during the 2024 academic year, spanning from the fourth year through the internship year. In the Iranian medical education system, the first three years are dedicated to basic and preclinical sciences, followed by four years of clinical training. The fourth year marks the start of core clinical rotations (e.g., internal medicine, surgery, pediatrics), during which students primarily observe and participate under close supervision. Subsequent years involve progressively greater clinical responsibility and patient contact, culminating in the internship year (typically the seventh year), which serves as a final, intensive clinical practicum with increased autonomy before graduation. Students at any point within this clinical phase (fourth year to internship) were eligible for inclusion. The inclusion criteria were being a currently enrolled clinical-phase medical student at ARUMS and providing written informed consent. Students who were on a formal leave of absence (e.g., medical, academic) during the data collection period were excluded.

Regarding academic assessment, ARUMS employs a norm-referenced grading system, in which student performance is evaluated relative to peers, fostering a highly competitive educational environment where grades are often curved. For international readers, it should be noted that the Grade Point Average (GPA) is reported on a 0–20 scale, with higher scores indicating better academic performance, as presented in the demographic characteristics (Table 1). This grading context is important for interpreting findings related to academic performance and perceived competition.

**Table 1:** Demographic characteristics of the participants (*n* = 237).

Variable	Categories	Frequency (n)	Percentage (%)
Age (years)	18–22	75	31.6
	23–27	162	68.4
GPA	12–14	30	12.7
	15–17	120	50.6
	18–20	87	36.7
Gender	Male	120	50.6
	Female	117	49.4
Marital status	Single	200	84.4
	Married	37	15.6
Previous mental health training	Yes	80	33.8
	No	157	66.2

**Fig. 2:**
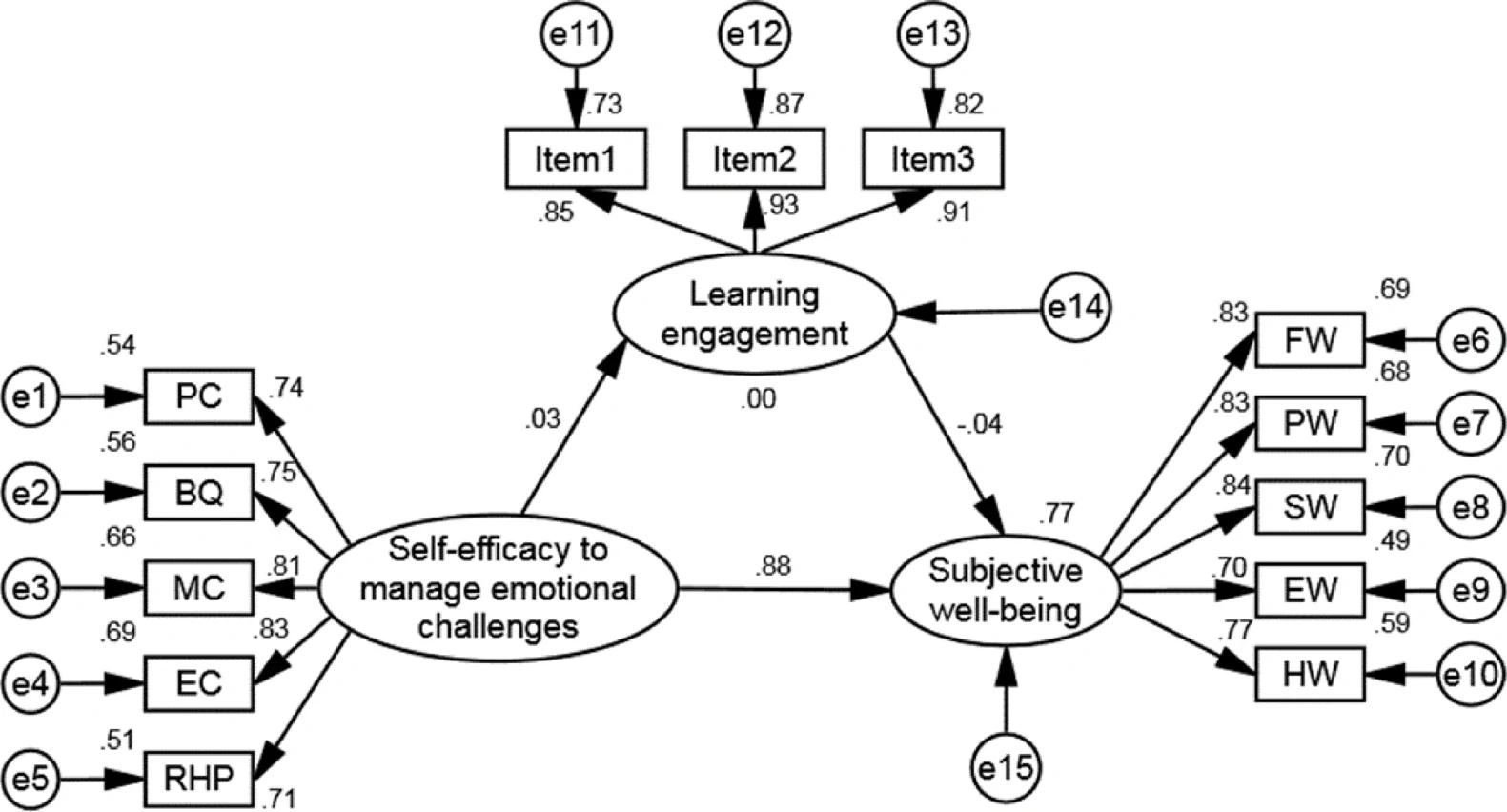
The path diagram (*n* = 237).

The required sample size was calculated using G*Power software (Version 3.1). For a multiple linear regression analysis assuming a medium effect size (f² = 0.15), an alpha level of 0.05, statistical power of 95%, and eight predictors, the minimum required sample size was estimated to be 209 participants. To compensate for potential non-response and missing data, the target sample size was increased by approximately 20%, resulting in an intended sample of 251 students. Accordingly, 251 questionnaires were distributed using a convenience sampling approach. Of these, 237 questionnaires were fully completed and returned, yielding a response rate of 94.4%. Fourteen questionnaires (5.6%) were excluded due to excessive missing data (i.e., more than 20% of items unanswered). In the final analytical sample (*n* = 237), no item-level missing data were present. Therefore, all analyses in SPSS and AMOS were conducted using complete-case (listwise) data, and no imputation procedures were required. The sample size reported in Fig. 2 was corrected to reflect the final analytical sample of 237 participants, and all reported degrees of freedom were rechecked to ensure consistency with this sample size.

### Instrument

#### Socio-demographic information form

This form was designed to collect demographic information based on relevant literature. It comprises six closed-ended questions, including age, gender, marital status, academic performance, and previous mental health training.

#### Physician self-efficacy to manage emotional challenges (PSMEC) scale

The Physician Self-efficacy to Manage Emotional Challenges (PSMEC) scale, created by Weurlander et al.^1^, comprises 17 items organized into five subscales. The subscales include: Subscale 1 (Patient Communication), with items 3, 5, 9, 13, 14, and 17; Subscale 2 (Being Questioned), with items 7 and 10; Subscale 3 (Medical Competence), featuring items 1, 4, 8, and 12; Subscale 4 (Educative Competence), which includes items 2, 11, and 15; and Subscale 5 (Relationships with Healthcare Professionals), encompassing items 6 and 16. Participants rated each item on a six-point Likert-type scale, ranging from 1 (strongly disagree) to 6 (strongly agree). The potential minimum and maximum scores for the scale are 17 and 102, respectively. The overall scale demonstrated a Cronbach’s alpha of 0.912, indicating high reliability. In this study, the Cronbach’s alpha of the total scale was reported to be 0.902, and its subscales were 0.828 and 0.886.

#### Learning engagement scale

Learning engagement was measured using a 3-item version of the Utrecht Work Engagement Scale^29^. This brief scale captures the core aspects of engagement in academic contexts through three key statements that participants rated on a 7-point frequency scale from 0 (never) to 6 (always). The selected items maintain the original scale’s conceptual foundation while being optimized for efficient administration in student populations. In the current study, this 3-item scale demonstrated good reliability, with a Cronbach’s alpha of 0.875 indicating strong internal consistency.

#### Well-being scale (WeBS)

The subjective well-being of participants was assessed using the Well-Being Scale (WeBS), originally developed by Lui and Fernando^30^. This multidimensional instrument comprises 29 items designed to measure five distinct domains: financial well-being (4 items: 2, 3, 4, 5), physical well-being (6 items: 1, 6, 7, 8, 9, 16), social well-being (4 items: 11, 12, 13, 18), eudaimonic well-being (7 items: 20, 21, 22, 23, 24, 25, 26), and hedonic well-being (3 items: 27, 28, 29). Responses are recorded on a six-point Likert-type scale ranging from 1 (strongly disagree) to 6 (strongly agree), resulting in potential total scores from 29 to 174, where higher scores indicate a greater level of subjective well-being. In its original validation, the scale demonstrated high internal consistency, with a Cronbach’s alpha of 0.92 for the total score. In this study, the Cronbach’s alpha of the total scale was reported to be 0.940, and its subscales were 0.830 and 0.920.

### Data analysis

Data were analyzed using IBM SPSS Statistics for Windows version 26.0 (IBM Corporation, Armonk, NY, USA) and AMOS version 24. Descriptive statistics (frequency, percentage, mean, standard deviation, median, and interquartile range) were used to summarize demographic characteristics and the main study variables. The normality of data distribution was assessed using the Kolmogorov-Smirnov test.

Pearson’s correlation coefficient was used to assess the bivariate relationships among the study variables. A hierarchical linear regression analysis was conducted to examine predictors of subjective well-being. In the regression models, the independent variables were self-efficacy to manage emotional challenges and learning engagement, while the dependent variable was subjective well-being. The assumptions of linear regression were thoroughly examined, including the assessment of multicollinearity using Variance Inflation Factor (VIF) and tolerance statistics, with VIF < 10 and tolerance > 0.1 indicating no substantial multicollinearity. The independence of errors was verified using the Durbin-Watson statistic, with values between 1.5 and 2.5 considered acceptable^31^.

To test the hypothesized mediation model, path analysis was employed using AMOS software. The mediation effect of learning engagement in the relationship between self-efficacy and subjective well-being was examined using bootstrapping procedures with 5000 resamples and bias-corrected 95% confidence intervals. Model fit was assessed using multiple indices: Chi-square/df ratio (< 3 acceptable), Comparative Fit Index (CFI > 0.90), Tucker-Lewis Index (TLI > 0.90), and Root Mean Square Error of Approximation (RMSEA < 0.08)^32^.

## Results

### Characteristics of the participants

A total of 237 medical students participated in the study. The demographic characteristics of the sample are summarized in Table 1.

### Descriptive statistics

Descriptive statistics for the primary study variables are presented in Table 2. Participants reported generally high scores on measures of self-efficacy in managing emotional challenges, learning engagement, and subjective well-being.

**Table 2:** Descriptive statistics of the main variables of the study (n = 237).

Variables	No. of items	Possible range	Observed range	Mean ± SD	Median (IQR)
Self-efficacy to manage emotional challenges (Total)	17	17–102	45–98	84.7 ± 11.8	86 (77–92)
Patient communication	6	1–36	12–35	28.9 ± 5.4	29 (25–33)
Being questioned	2	2–12	3–11	7.7 ± 1.8	8 (7–9)
Medical competence	4	4–24	10–24	19.1 ± 3.2	20 (17–22)
Educative competence	3	3–18	6–18	14.3 ± 2.7	14 (12–17)
Relationships with healthcare professionals	2	2–12	5–12	10.2 ± 1.7	10 (9–12)
Learning engagement (Total)	3	0–18	5–18	13.8 ± 3.1	14 (12–16)
Subjective well-being (Total)	29	29–174	89–165	132.6 ± 16.4	135 (122–146)
Financial well-being	4	4–24	7–23	14.5 ± 3.9	14 (12–17)
Physical well-being	6	6–36	18–35	26.8 ± 4.2	27 (24–30)
Social well-being	4	4–24	13–24	19.7 ± 2.8	20 (18–22)
Eudemonic wellbeing	7	7–42	21–41	33.5 ± 4.3	34 (30–37)
Hedonic well-being	3	3–18	8–18	13.2 ± 2.6	13 (11–15)

SD: Standard Deviation; IQR: Interquartile Range.

### Correlation analysis

As shown in Table 3, Pearson correlation analyses revealed significant positive correlations among all primary study variables (self-efficacy to manage emotional challenges, learning engagement, and subjective well-being).

**Table 3:** Correlation matrix of the main variables of the study (n = 237).

Variables	1	2	3
	r (p)		
1. Self-efficacy to manage emotional challenges	1		
2. Learning engagement	0.521 (p < 0.001^*^)	1	
3. Subjective well-being	0.586 (p < 0.001^*^)	0.513 (p < 0.001^*^)	1

Numbers 1–3 in the title row represent the numbered variables in the first column.

^*^Correlation is significant at the 0.05 level (2-tailed).

### Regression analysis

A hierarchical linear regression was conducted to examine predictors of subjective well-being (Table 4). The initial model, which included only demographic variables, accounted for a small but significant portion of the variance. The addition of self-efficacy to manage emotional challenges and learning engagement in the second block resulted in a substantial, statistically significant increase in explained variance. In the final model, both self-efficacy and learning engagement were significant positive predictors of subjective well-being.

**Table 4:** The hierarchical linear regression analysis coefficients to examine predictors of the subjective well-being (n = 237).

Variables	B	SE	Beta	*p*-value	95% CI for B	
					LB	UB
Block 1: Demographic variables						
Age (18–22 = 0^a^)	1.521	1.213	0.061	0.211	-0.868	3.901
GPA (12–14 = 0^a^)						
15–17	2.158	1.856	0.078	0.247	– 1.492	5.794
18–20	3.981	2.011	0.126	0.049	0.021	7.945
Gender (Male = 0^a^)	– 1.209	1.458	– 0.044	0.409	– 4.056	1.660
Marital status (Single = 0^a^)	3.109	2.108	0.075	0.140	– 1.05	7.235
Previous mental health training (Yes = 0^a^)	1.850	1.501	0.062	0.219	– 1.102	4.809
Model summary	R^2^ = 0.05, F _(6, 243)_ = 2.189, *p* < 0.001					
Block 2: SMEC and LE						
Self-efficacy to manage emotional challenges	0.653	0.086	0.455	< 0.001	0.492	0.813
Learning engagement	1.557	0.313	0.317	< 0.001	0.965	2.144
Model summary	R^2^ = 0.480, ΔR^2^ = 0.43, ΔF _(2, 241)_ = 98.158, *p* < 0.001, DW = 1.980					

B Unstandardized coefficient; SE Standard error; Beta Standardized coefficient; LB Lower bound; UB Upper bound; DW Durbin-Watson test. ^a^ Reference groups.

To evaluate the robustness of the primary findings, the final regression model (Block 2) was also analyzed without including demographic covariates. The results from this model remained essentially unchanged. Both self-efficacy in managing emotional challenges and learning engagement remained significant positive predictors of subjective well-being, with standardized coefficients (βs) and levels of statistical significance similar. The overall variance explained (R²) was nearly identical. These results indicate that the relationships between the key psychological constructs are strong and not dependent on the demographic variables considered in this study.

### Path analysis

Path analysis was used to test the proposed mediation model. The model demonstrated a good fit to the data. The results (Table 5; Fig. 2) supported the hypotheses, indicating a significant total effect of self-efficacy on well-being and a significant indirect effect through learning engagement. The direct impact also remained important, confirming partial mediation.

**Table 5:** Direct, indirect, and total effects from the mediation analysis testing learning engagement as a mediator (n = 237).

Effect type	Pathway	Effect	SE	Estimated (β)	BC 95% CI	
					LL	UL
Total effect	c	0.852	0.092	0.595^*^	0.677	1.032
Indirect effect	ab	0.213	0.055	-	0.115	0.319
Direct effect	cʹ	0.657	0.089	0.450^*^	0.492	0.818

BC Bias-Corrected Bootstrap Lower and Upper-Level Confidence Intervals (5000 samples), CI Confidence Interval.

^*^Significant at the 0.01 level (2-tailed).

A sensitivity analysis was performed to evaluate the impact of including demographic covariates such as age, gender, GPA, marital status, and prior mental health training on the primary findings of the mediation model. The path analysis was then repeated without these covariates. The results demonstrated that both the pattern and significance of all direct and indirect effects remained essentially unchanged. The point estimates for the total effect (path c), the indirect effect (ab), and the direct effect (c’) of self-efficacy on well-being, along with the model fit indices, closely align with those reported in the initial analysis (refer to Table 5). The proposed mediation pathway through learning engagement is a robust finding, remaining consistent across the specific demographic characteristics included in the primary model.

## Discussion

The demanding nature of medical education, marked by significant academic pressure and emotional challenges, underscores the importance of identifying factors associated with medical students’ subjective well-being. This study investigated the relationships between emotional management self-efficacy, learning engagement, and subjective well-being. A key finding was the significant association between higher academic performance (GPA) and greater subjective well-being among participants. This finding should be interpreted within the specific context of the norm-referenced grading system at ARUMS, where performance is evaluated relative to peers, inherently fostering a competitive environment. While this finding aligns with research linking academic achievement to feelings of competence^24,33^, it is essential to interpret it cautiously. The relationship between GPA and well-being is likely complex and bidirectional. Academic success may contribute to a sense of accomplishment and well-being, but conversely, higher baseline well-being or supportive learning conditions may also facilitate academic performance^34^.

Furthermore, an exclusive focus on grades can sometimes lead to stress and burnout^35,36^. Therefore, this association highlights the potential benefit of educational approaches that promote mastery and competence while mitigating excessive grade-focused competition, potentially fostering both academic success and psychological health. A shift toward a criterion-referenced (standards-based) grading system, where grades reflect the achievement of predefined learning objectives rather than peer comparison, could be a strategic intervention. Such a system may help reduce excessive competition, promote a mastery-oriented learning climate, and potentially support both academic success and psychological health^37^.

Our study demonstrates a significant association between emotional management self-efficacy (EMSE) and subjective well-being (SWB) among medical students, suggesting that EMSE is a crucial factor for psychological well-being in this population. This finding is consistent with broader research indicating that EMSE correlates with well-being through various pathways, such as its association with lower psychological distress via better interpersonal adaptation^38^and its role as a factor linked to lower levels of professional burnout among healthcare professionals^39,40^. In the context of Iran’s demanding medical education system, which involves intense academic competition and challenging transitions^41,42^, fostering EMSE appears particularly relevant for student adaptation. The observed relationship is also supported by studies showing self-efficacy as a factor associated with the link between meaning in life and SWB^43,44^. However, the cross-sectional design of our study precludes causal inferences. It is equally plausible that students with higher baseline well-being develop greater confidence in managing emotions, or that other unmeasured variables (e.g., personality traits like neuroticism, pre-existing coping skills, or quality of social support) influence both EMSE and SWB. Therefore, while our results highlight an essential association, future longitudinal research is needed to clarify the directionality of this relationship and account for potential confounding variables.

Our findings suggest that learning engagement is significantly associated with medical students’ subjective well-being. A robust body of educational research, including a systematic review by Wong et al., reports a consistent positive correlation between student engagement and subjective well-being^19^. This relationship is critical, as engagement has been identified as a factor related to persistence, whereas low well-being and motivation are associated with higher dropout intentions^45^. Engaged students may experience enhanced well-being through mechanisms such as increased belonging and self-esteem^20,46^, and digital learning platforms can foster a sense of ‘flow’ that supports both engagement and well-being^47^. Furthermore, environmental factors such as social support and a sense of community are strongly associated with engagement levels^48^. Significantly, well-being itself is associated with better academic performance^34^, suggesting a potential reciprocal relationship. Therefore, engagement may act as a psychological resource that coincides with greater resilience and well-being. However, the direction of this association in our study remains unclear; students with higher well-being may be more able to become engaged in their learning. Medical education systems should consider promoting conditions conducive to engagement, such as active learning, mentorship, and supportive communities, not as a guaranteed causal intervention, but as a strategy aligned with factors that co-occur with student wellness.

A central finding of this study is the significant mediating role of learning engagement in the association between emotional management self-efficacy (EMSE) and subjective well-being. This serial relationship aligns with theoretical models in educational psychology, such as the framework proposed by Yin et al.^20^, which posits that self-efficacy and engagement can function as sequential mediators. The importance of engagement, a factor closely linked to well-being, is supported by meta-analytic evidence^19^. Furthermore, learning environments that support autonomy and competence are associated with both higher engagement and well-being^49^, and emotion regulation ability, a key component of EMSE, is significantly correlated with academic engagement^50^. In the demanding context of medical education, where a lack of personal resources is linked to burnout and engagement is considered a protective factor^51^, understanding these interrelated constructs is valuable.

### Limitations

This study boasts several strengths, notably its innovative exploration of learning engagement as a mediator between self-efficacy in managing emotional challenges and subjective well-being among medical students. The application of a comprehensive theoretical framework, the use of validated instruments, and an impressive response rate of 94.4% further bolster its validity. However, there are several limitations that necessitate cautious interpretation of the findings. The cross-sectional design restricts the ability to establish causal relationships. While the proposed model indicates a pathway from self-efficacy to well-being through engagement, it is important to acknowledge the possibility of reverse or bidirectional effects. Additionally, unmeasured confounding variables, such as personality traits and prior mental health history, may account for the observed associations.

Moreover, the inclusion of students from different stages of clinical training may introduce variability in experience and stress levels that our analysis did not address. The reliance on convenience sampling from a single university limits the generalizability of the results, and the use of self-reported measures might introduce bias. Furthermore, the unique cultural and educational context of Iran complicates the applicability of the findings to other settings. Future research should employ longitudinal or experimental designs, account for confounding variables, investigate the moderating role of the training stage, and replicate the study across various cultural contexts to validate the proposed model.

### Implications

The findings of this study carry essential practical and theoretical implications. Theoretically, identifying learning engagement as a partial mediator enriches our understanding of the psychological mechanisms linking self-efficacy to subjective well-being in the academic context of medical education. It suggests that the positive effect of emotional self-efficacy on well-being is not only direct but also mediated by increased dedication and involvement in learning activities.

Practically, these results underscore the importance of developing targeted interventions within medical schools. Educational programs and support services should aim not only to enhance students’ self-efficacy in managing emotional challenges but also to foster their active engagement in the learning process. Strategies could include incorporating resilience training, promoting interactive and meaningful learning environments, and providing psychological support to strengthen students’ personal and academic resources. By addressing both constructs, institutions can more effectively promote medical students’ overall well-being, which is crucial to their personal development and future professional performance.

## Conclusion

This study highlights the critical role of self-efficacy in managing emotional challenges and its positive influence on learning engagement and subjective well-being among medical students. The findings indicate that enhancing self-efficacy not only promotes greater learning engagement but also significantly contributes to students’ overall psychological health. By establishing learning engagement as a mediating factor, the research offers valuable insights for educational planners and policymakers seeking to improve the well-being of medical students. Implementing targeted interventions that cultivate emotional management skills and encourage active learning can enhance academic performance and resilience within medical education. Future research should strive to replicate these findings across diverse cultural contexts and employ longitudinal designs to clarify the causal pathways involved, ultimately aiding the development of more effective support systems for medical students.

## Data Availability

The data that support the findings of this study are available from the corresponding author, [Mahzad Yousefian], upon reasonable request.

## Abbreviations

ARUMS: Ardabil university of medical sciences
CFI: Comparative fit index
CI: Confidence interval
DW: Durbin-watson
EMSE: Emotional management self-efficacy
GPA: Grade point average
LE: Learning engagement
PSMEC: Physician self-efficacy to manage emotional challenges
RMSEA: Root mean square error of approximation
SD: Standard deviation
SE: Standard error
SWB: Subjective well-being
TLI: Tucker-Lewis Index
VIF: Variance inflation factor
WeBS: Well-being scale
